# Positive result of Sars‐Cov‐2 in faeces and sputum from discharged patients with COVID‐19 in Yiwu, China

**DOI:** 10.1002/jmv.25905

**Published:** 2020-07-28

**Authors:** Youjiang Li, Yingying Hu, Yuanyuan Yu, Xiaodong Zhang, Bin Li, Jianguo Wu, Junyu Li, Yingping Wu, Xiaoping Xia, Huina Tang, Jian Xu

**Affiliations:** ^1^ The Department of Cinical Laboratory, The Fourth Affiliated Hospital Zhejiang University School of Medicine Yiwu Zhejiang China; ^2^ The Department of Obstetrics and Gynecology, The Fourth Affiliated Hospital Zhejiang University School of Medicine Yiwu Zhejiang China; ^3^ The Department of Infectious Diseases, The Fourth Affiliated Hospital Zhejiang University School of Medicine Yiwu Zhejiang China; ^4^ The Department of Radiology, The Fourth Affiliated Hospital Zhejiang University School of Medicine Yiwu Zhejiang China

**Keywords:** COVID‐19, discharge criteria, SARS‐CoV‐2, virus nucleic acid test

## Abstract

**Background:**

With the effective prevention and control of COVID‐19 in China, the number of cured cases has increased significantly. Further monitoring of the disease prognosis and effective control of the “relapse” of the epidemic has become the next focus of work. This study analysed the clinical prognosis of discharged COVID‐19 patients by monitoring their SAR‐CoV‐2 nucleic acid status, which provided a theoretical basis for medical institutions to formulate discharge standards and follow‐up management for COVID‐19 patients.

**Methods:**

We included 13 discharged COVID‐19 patients who were quarantined for 4 weeks at home. The patient's daily clinical signs were recorded and sputum and faecal specimens were regularly sent for detection of SARS‐CoV‐2 nucleic acid.

**Results:**

The time between initial symptoms and meeting discharge criteria was 18 to 44 days with an average of 25 ± 6 days. The faecal samples of two patients still tested positive after meeting the discharge criteria and the sputum samples of four patients returned positive 5 to 14 days after discharge. The rate of the recurring positive test result in samples from the respiratory system was 31% (4/13).

**Conclusion:**

Under the present discharge criteria, the high presence of SARS‐CoV‐2 nucleic acid in faecal and respiratory samples of discharged COVID‐19 patients indicates potential infectivity. Therefore, we suggest that faecal virus nucleic acid should be tested as a routine monitoring index for COVID‐19 and a negative result be added to the criteria. Simultaneously, we should strengthen the regular follow‐up of discharged patients with continuous monitoring of the recurrence of viral nucleic acid.

## Highlights

- The positive rate of SARS‐CoV‐2 nucleic acid in faeces and respiratory tract of discharged COVID‐19 patients both were high, which indicate potential infectivity.

## 1 INTRODUCTION

In December 2019, a novel coronavirus caused an outbreak in Wuhan, China.^1^, ^2^ The World Health Organization (WHO) declared the novel coronavirus pneumonia (COVID‐19) outbreak to be a Public Health Emergency of International Concern (PHEIC) on January 30, 2020 and a global pandemic on March 11, 2020.^3^ By March 28, 2020, SARS‐CoV‐2 had spread to more than 150 countries worldwide with 81 439 infections and 75 448 discharged in China alone.^4^

SARS‐CoV‐2 belongs to a genus of coronaviruses that are enveloped with round or oval particles 60 to 140 nm in diameter and are often polymorphic. COVID‐19 is genetically different from SARS‐CoV and MERS‐CoV.^5^ Current studies show that the homology of coronavirus with bat SARS is more than 85%. At present, researchers focus on the epidemiological history, clinical characteristics, treatment plan, and virus detection methods of confirmed cases and less attention has been given to the clinical outcome, follow‐up visits, and management of discharged patients. Recently, it was reported in many places in China that the nucleic acid test results of discharged patients returned positive during their follow‐up visits. However, there are no available studies on the causes, clinical symptoms, secondary infection, and timeline of viral nucleic acid re‐emergence in these patients. With effective prevention and control of COVID‐19 in China, the number of cured cases has increased significantly. Further monitoring of the disease prognosis and effective control of the “relapse” of the epidemic has become the next focus of work. As one of the designated hospitals for the diagnosis and treatment of COVID‐19, all patients in our hospital met the discharge criteria after treatment as described in "Diagnosis and Treatment Scheme of New Coronavirus Infected Pneumonia (trial version 5)”.^6^ The aim of this study is to analyse the causes of viral nucleic acid re‐emergence by investigating the follow‐up data of COVID‐19 patients who met the discharge criteria. This may provide evidence that medical institutions should strengthen the follow‐up management of discharged COVID‐19 patients.

## 2 MATERIALS AND METHODS

### 2.1 Study population

We recruited 13 patients who were diagnosed as COVID‐19 at the Fourth Affiliated Hospital of Zhejiang University from January 26, 2020 to February 6, 2020. All patients with COVID‐19 tested positively for SARS‐CoV‐2 by the use of quantitative reverse‐transcription polymerase chain reaction (RT‐PCR) on samples from the respiratory tract. All cases met the latest discharge criteria or COVID‐19 patients from February 9, 2020 to February 28, 2020. Discharge criteria of COVID‐19 were based on the Diagnosis and Treatment Scheme of New Coronavirus Infected Pneumonia: (a) normal body temperature more than or equal to 3 days, (b) resolved respiratory symptoms, (c) substantially improved acute exudative lesions on chest computed tomography (CT) images, and (d) two consecutively negative viral nucleic acid test results at least 1 day apart in specimens from the respiratory tract. This study was reviewed and approved by the Medical Ethical Committee of the Fourth Affiliated Hospital, Zhejiang University School of Medicine (approval number K20200025). Written informed consent was obtained from each enrolled patient.

### 2.2 Follow‐up observation and monitoring indicators

Oral swabs, nasal swabs, sputum, blood, faeces, urine, vaginal secretions, and milk of COVID‐19 patients were collected for viral nucleic acid test upon hospitalisation. After COVID‐19‐confirmed patients meet the discharge criteria, they should: (a) Be quarantined for 4 weeks at home (b) wear medical masks when leaving home and keep distance with others to reduce contact (c) attend a follow‐up visit in the first, second, and fourth week after discharge with sputum and faecal viral nucleic acid tests, routine blood examination, biochemistry tests including C‐reactive protein, and lung CT scan. (d) Monitor body temperature and other symptoms daily during isolation. If any symptoms such as fever, cough, sore throat, fatigue, and muscle soreness are found, patients should immediately return to the hospital to determine the respiratory and faecal virus nucleic acid status.

### 2.3 Viral nucleic acid test

SARS‐CoV‐2 nucleic acid was detected by real‐time RT‐PCR. The nucleic acid RT‐PCR test kit we employed was recommended by the Chinese Center for Disease Control and Prevention (China CDC) and produced by Shanghai Zhijiang Biotechnology, China. Nucleic acid was extracted from the samples using an EX2400 Automatic Nucleic Acid Extractor (Shanghai Zhijiang Biotechnology). RT‐PCR was performed in a LightCycler 480 Instrument II (Roche, Germany). We quantified the RdRP, E, and N gene in the SARS‐CoV‐2 genome and used the criteria described by the manufacturer to determine positivity. Specimen collection, treatment, and laboratory procedures were in strict accordance with the operating procedures recommended by WHO for RT‐PCR, and we performed clinical resampling if results were unclear.

### 2.4 Data acquisition

Baseline information, laboratory and imaging examinations, diagnosis, and treatment process of the included cases were obtained from the electronic medical record system. All information was obtained and curated with a standardised data collection form. Two researchers independently reviewed the data collection forms.

### 2.5 Statistical analysis

Statistical analysis was performed using SPSS 20.0. Continuous variables were summarised as means and standard deviations or medians and interquartile ranges as appropriate. Categorical variables were expressed as counts and percentages in each category. For laboratory results, we also assessed whether the measurements were outside the normal range.

## 3 RESULTS

### 3.1 Demographics and baseline characteristics of COIVD‐19 patients

We included 13 patients confirmed with COVID‐19, six males and seven females with an average age of 52.8 ± 20.2 years. The time from onset of symptoms to the diagnosis of the COVID‐19 patients was 1 to 19 days and discharge criteria were met between 9 and 28 February 2020. The time from the initial onset of symptoms to meeting the discharge criteria was 18 to 44 days with a median time of 25 ± 6 days. According to the epidemiological history of the patients, four were long‐term residents of Wuhan returning to Yiwu, nine had direct contact with people who had returned from Wuhan. All patients presented with common main symptoms of fever, cough, fatigue, muscle soreness, and sore throat. The laboratory parameters showed that 10 cases had a low to normal white blood cell count, five cases had lymphocytopenia, and only two cases presented with elevated C‐reactive protein while all 13 cases had normal transaminase levels. The CT examination indicated the typical features of viral pneumonia such as patchy ground‐glass opacity. The main therapies were antiviral, antibiotic, and oxygen inhalation without additional glucocorticoid treatment (Table 1).

**Table 1:** Clinical and laboratory characteristics of patients with COVID‐19

Clinical characteristics	Patient 1	Patient 2^b^	Patient 3^b^	Patient 4	Patient 5^a^	Patient 6	Patient 7	Patient 8^b^	Patient 9^b^	Patient 10	Patient 11	Patient 12	Patient 13^a^
Sex	Female	Female	Female	Female	Female	Female	Female	Male	Male	Male	Male	Male	Male
Age, y	24	72	71	56	33	37	33	37	73	47	1	70	22
Other family members infected	No	Yes	No	Yes	No	Yes	Yes	Yes	Yes	Yes	Yes	Yes	Yes
Date of first symptom	Jan. 23	Jan. 23	Jan. 20	Jan. 22	Jan. 12	Jan. 23	Jan. 30	Jan. 23	Jan. 15	Jan. 11	Jan. 30	Jan. 31	Jan. 22
Date of admission	Feb. 3	Jan. 30	Feb. 6	Jan. 28	Feb. 28	Jan. 26	Feb. 2	Jan. 26	Jan. 28	Jan. 30	Feb. 1	Feb. 1	Jan. 29
Date of meeting discharge criterion	Feb. 17	Feb. 19	Feb. 12	Feb. 11	Feb. 9	Feb. 11	Feb. 17	Feb. 14	Feb. 9	Feb. 24	Feb. 28	Feb. 28	Feb. 10
Days between initial symptoms and meeting discharge criteria	23	27	23	20	27	19	18	22	25	44	30	28	19
Inpatient days	14	19	6	14	11	16	15	19	12	25	27	27	12
Sputum SARS‐CoV‐2 negative to positive (days)	NA	14	7	NA	NA	NA	NA	5	6	NA	NA	NA	NA
Days of faecal SARS‐CoV‐2 positive after sputum turning negative	NA	NA	NA	NA	14	NA	NA	NA	NA	NA	NA	NA	15
Complications	None	Chronic hypertension	Chronic hypertension	Chronic hypertension	None	None	Depression	None	Chronic	None	None	None	None
									hypertension				
									Alcoholic liver disease				
		Chronic lymphocytic leukemia	Old tuberculosis	Depression									
Signs and symptoms													
Fever	Yes	Yes	Yes	Yes	Yes	No	No	Yes	Yes	Yes	Yes	No	Yes
Cough	No	Yes	Yes	Yes	Yes	No	No	Yes	Yes	Yes	No	Yes	Yes
Myalgia	No	No	No	No	No	No	Yes	Yes	No	Yes	No	No	No
Fatigue	No	No	Yes	No	No	No	Yes	Yes	No	No	No	No	No
Sore characteristics
White blood cell count (×10^9^/L)	3.5	30.9	4.2	5.7	5.7	4.3	4.6	5.8	5.5	4.4	7	4.8	5.6
Low or normal leukocyte count (<9.5 × 10^9^/L)	Yes	No	Yes	Yes	Yes	Yes	Yes	Yes	Yes	Yes	Yes	Yes	Yes
Lymphocyte count (×10^9^/L)	0.8	26.9	0.7	1.7	1.7	1.1	1.1	0.54	1.6	1.9	3.5	1.8	1.9
Lymphopenia (<1.5 × 10^9^/L)	Yes	No	Yes	No	No	Yes	Yes	Yes	No	No	No	No	No
Elevated ALT (>45 U/L) or AST (>35 U/L)	No	No	No	No	No	No	No	No	No	No	No	No	No
ALT (U/L)	23	19	18	9	18	17	18	21	8	15	8	21	18
AST (U/L)	34	25	19	30	23	17	23	23	15	17	111	19	18
C‐reactive protein (Normal: <6 mg/L)	2.3	3.6	50.2	19.5	0.1	0.7	0.1	2.4	1.2	4.7	0.6	0.5	0.4
CT evidence of pneumonia													
Typical signs of viral infection	Yes	Yes	Yes	Yes	Yes	Yes	Yes	Yes	Yes	Yes	Yes	Yes	Yes
Lung CT scans(discharged)	Inflammation absorption	Inflammation absorption	Inflammation absorption	Inflammation absorption	Inflammation absorption	Inflammation absorption	Inflammation absorption	Inflammation absorption	Inflammation absorption	Inflammation absorption	Inflammation absorption	Inflammation absorption	Inflammation absorption
Diagnosis (Clinical type)	Common type	Common type	Common type	Common type	Common type	Common type	Common type	Common type	Common type	Common type	Common type	Common type	Common type
Treatment													
Oxygen support (nasal catheter)	No	Yes	Yes	No	No	Yes	No	No	Yes	No	No	No	No
Antiviral therapy	Yes	Yes	Yes	Yes	Yes	Yes	Yes	Yes	Yes	Yes	No	Yes	Yes
Antibiotic therapy	No	Yes	No	Yes	No	Yes	No	Yes	No	No	No	No	Yes
Use of corticosteroid	No	No	No	No	No	No	No	No	No	No	No	No	No

*Note*: Diagnosis and discharge criteria of COVID‐19 was based on the New Coronavirus Pneumonia Prevention and Control Program (5th edition) published by the National Health Commission of China.

Abbreviations: ALT, alanine transaminase; AST, aspartate transaminase; COVID‐19, 2019 novel coronavirus disease; NA, not applicable; SARS‐CoV‐2, severe acute respiratory syndrome coronavirus 2.

a Patient that had continued positivity of SARS‐CoV‐2 nucleic acid in faeces after negative results from respiratory samples.

b Patient that had recurrence of SARS‐CoV‐2 nucleic acid in sputum after discharge.

### 3.2 Viral nucleic acid test results before discharge

**Figure 1:**
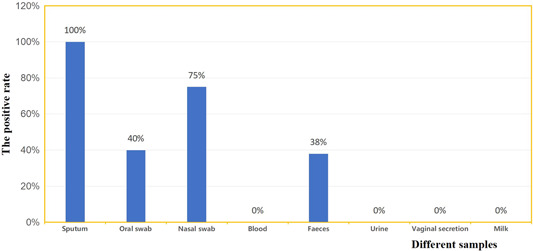
Positive rate of SARS‐CoV‐2 nucleic acid test in different types of specimens

We tested each type of specimen more than 3 times for SARS‐CoV‐2 nucleic acid upon hospitalisation. Test results showed that the positive rate in sputum was 100% (13/13), oral swab was 40% (4/10), nose swab was 75% (9/12), and faeces was 38% (5/13). Concurrently, the urine and blood viral nucleic acid of all 13 patients tested negative. Similarly, the vaginal secretions of the six female patients tested negative and the milk of one lactating patient was also negative (Table 2 and Figure 1).

**Table 2:** Results of SARS‐CoV‐2 nucleic acid test in different types of specimens of COVID‐19 patients

Patients	Sex	Age	Specimen							
			Sputum	OS	NS	Blood	Faeces	Urine	VS	BM
1	Female	24	+	−	+	−	−	−	−	NT
2	Female	72	+	−	+	−	−	−	−	NT
3	Female	71	+	NT	+	−	+	−	−	NT
4	Female	56	+	−	−	−	−	−	NT	NT
5	Female	33	+	−	+	−	+	−	−	NT
6	Female	37	+	−	−	−	−	−	−	NT
7	Female	33	+	+	+	−	−	−	−	−
8	Male	37	+	+	+	−	−	−	NT	NT
9	Male	73	+	NT	−	−	−	−	NT	NT
10	Male	47	+	−	+	−	−	−	NT	NT
11	Male	1	+	NT	NT	−	+	−	NT	NT
12	Male	70	+	+	+	−	+	−	NT	NT
13	Male	22	+	+	+	−	+	−	NT	NT
Total‐no./total no. (%)			13/13(100)	4/10(40)	9/12(75)	0/13(0)	5/13(38)	0/13(0)	0/6(0)	0/1(0)

Abbreviations: +, positive; −, negative; BM, breast milk; NT,  Do not detect; NS, nasal swab; OS, oral swab; VS, vaginal secretion.

#### 3.2.1 Follow‐up results of patients after meeting discharge criteria

**Figure 2:**
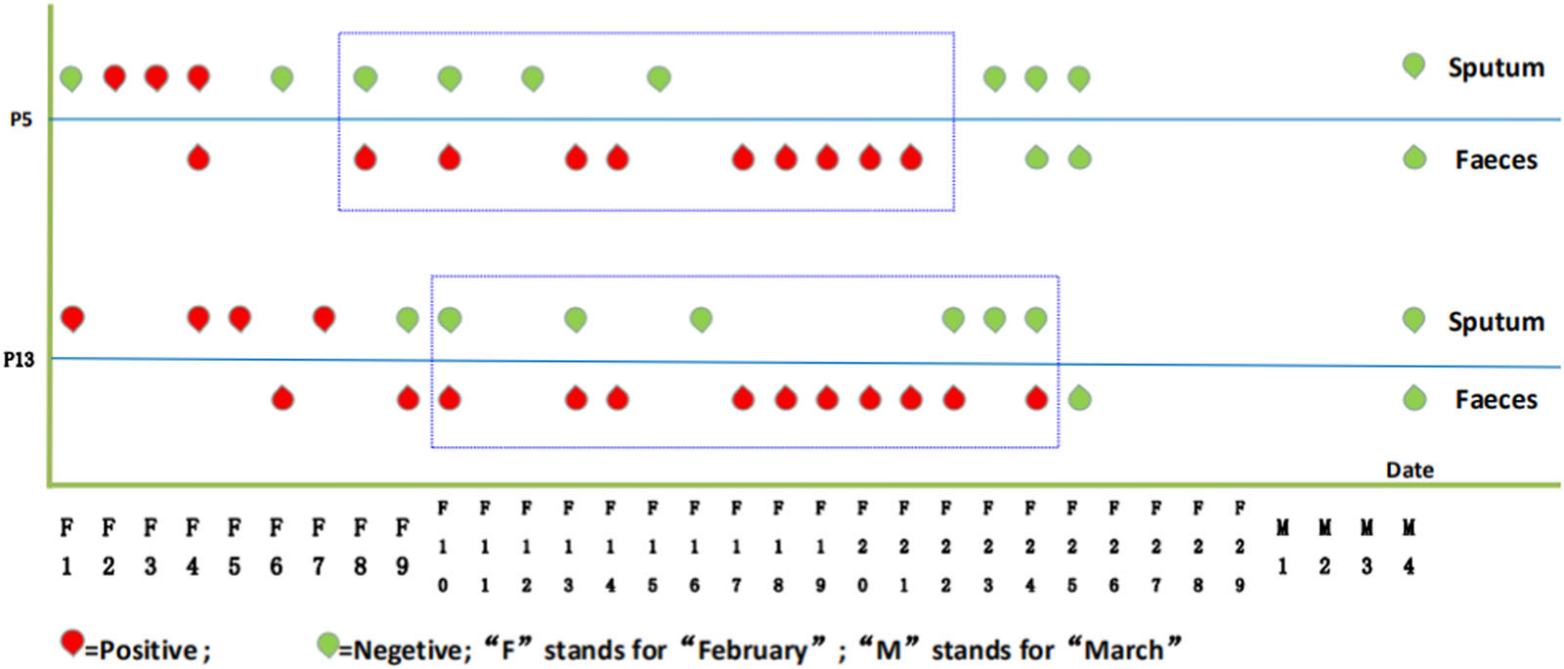
Continued positivity of SARS‐CoV‐2 nucleic acid in faeces of two patients after negative results from respiratory samples

In subsequent monitoring of the viral nucleic acid status of discharged patient, faeces from two patients (patient 5 and 13) continuously tested positive for SARS‐CoV‐2 even though the sputum was negative. The faeces from patient 5 tested positive for 14 days and patient 5 for 15 days after sputum was negative (Figure 2).

**Figure 3:**
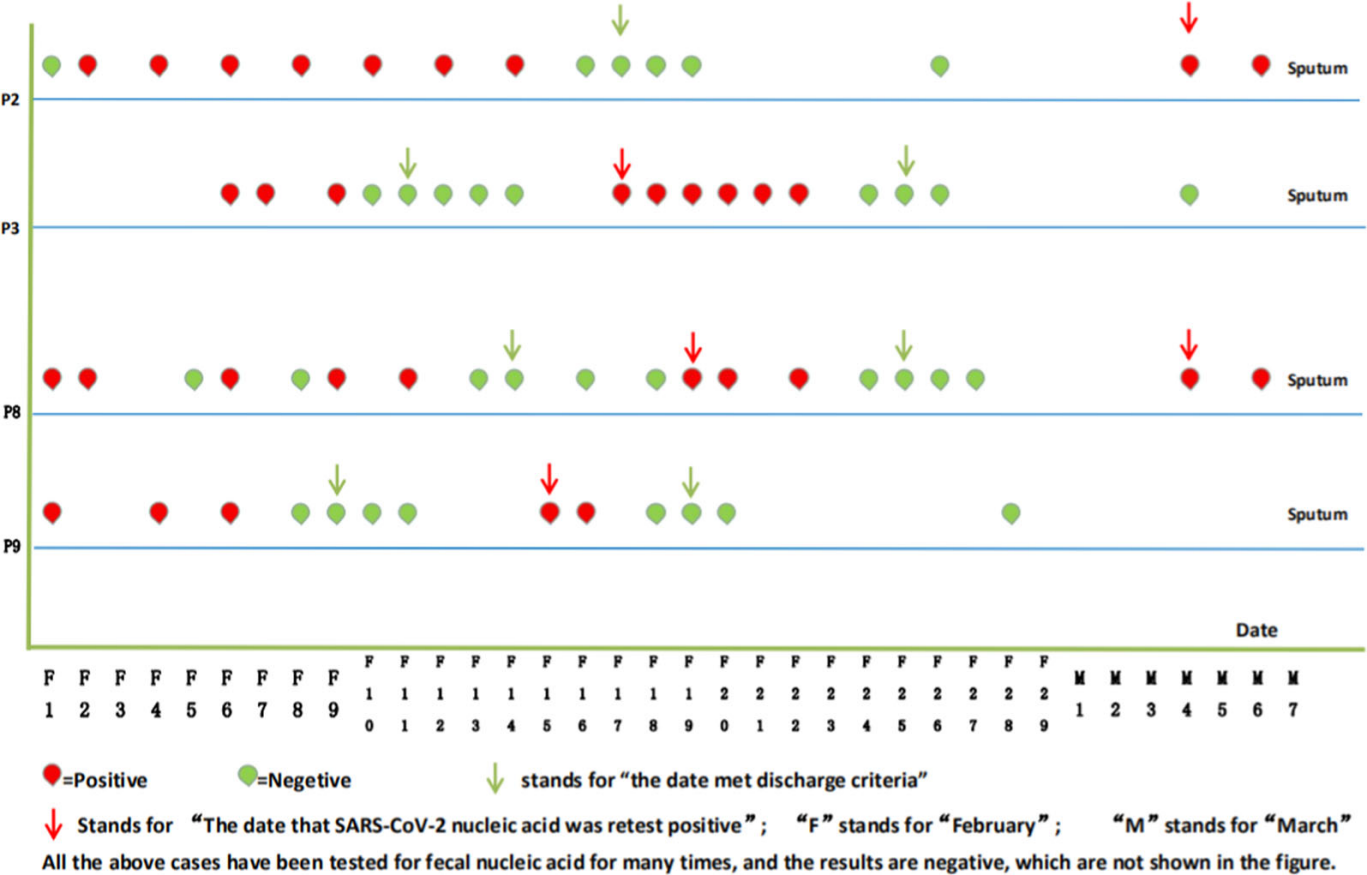
Recurrence of SARS‐CoV‐2 nucleic acid in four patients after discharge

**Figure 4:**
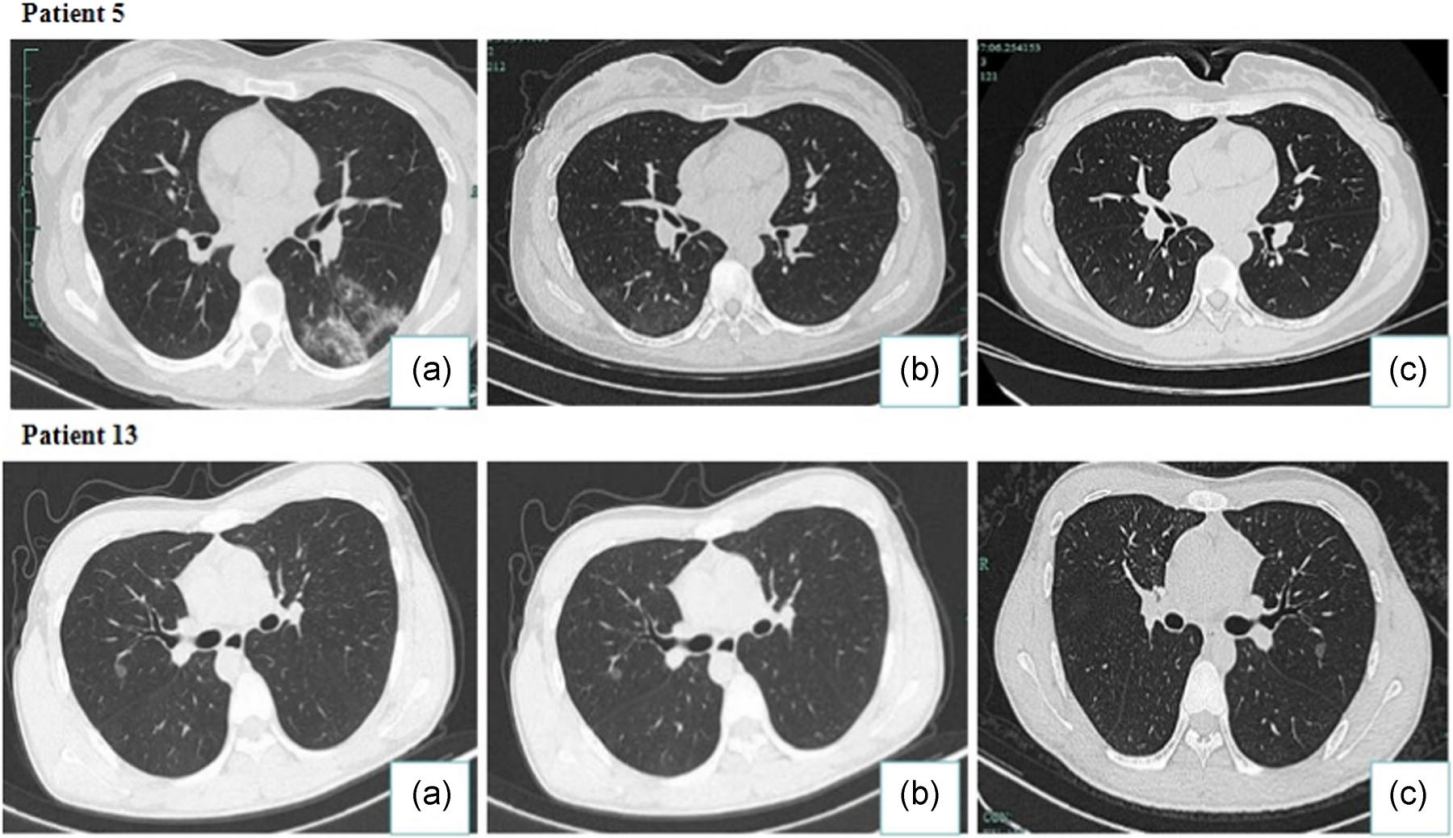
Transverse chest CT images from COVID‐19 patients with faecal SARS‐CoV‐2 nucleic acid positivity. A, During hospitalization, CT scans typical signs of COVID‐19: density‐increased patchy consolidation and ground‐glass shadow. B, Lung CT scan of patients meeting discharge criteria: strip high‐density shadows have been mostly absorbed. C, Lung CT scans at the time of faecal SARS‐CoV‐2 nucleic acid positivity after sputum turned negative: similar to (B). CT, computed tomography

**Figure 5:**
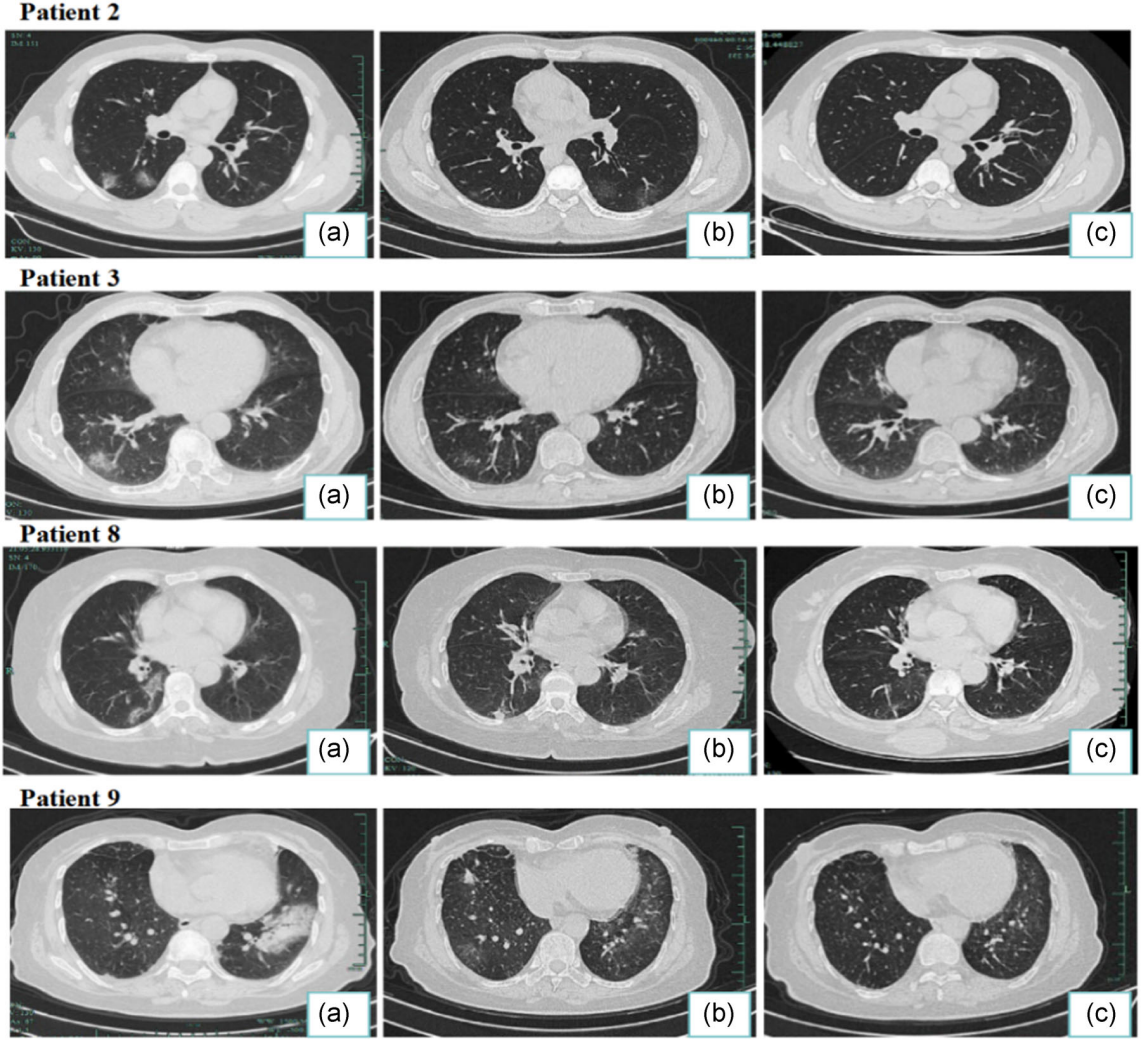
Transverse chest CT images from COVID‐19 patients with recurence of SARS‐CoV‐2. A, During hospitalization, CT scans typical signs of COVID‐19: density‐increased patchy consolidation and ground‐glass shadow. B, CT scans while patient had met the current discharge standards:strip high‐density shadows of the lung with largely absorbed. C, Lung CT scans at the time of nucleic acid recurence: similar to (B). CT, computed tomography

By March 28, 2020, all discharged patients were followed‐up for 4 weeks. Four discharged patients (Patient 2, 3, 8, 9) presented a positive sputum viral nucleic acid test during a later follow‐up between 5 and 14 days after discharge. In particular, patient 8 who had a recurrence of viral nucleic acid 5 days after discharge was able to meet discharge criteria again, after which the patient once more tested positive 7 days after the second discharge (Figure 3). And patient 2 had a complication of chronic lymphocytic leukemia (Table 1). Although we detected SARS‐CoV‐2 nucleic acid in these patients after discharge, the blood examination, C‐reactive protein levels, biochemistry, and lung CT showed no obvious abnormalities and no clinical symptoms were observed (Figures 4 and 5). The sputum and faecal samples of the other seven patients remained negative in absence of clinical symptoms. Similarly, the laboratory and imaging results showed no abnormalities.

## 4 DISCUSSION

Currently, the recognised transmission route of COVID‐19 is mainly through respiratory droplets and close contact. The patient's infectivity is determined by the presence of the virus in different body fluids, secretions, and excrements. It has been confirmed that viral nucleic acid can be detected in samples of oral swabs, sputum, faeces, urine, and tears of COVID‐19 patients.^7^, ^8^ In this study, 5 of the 13 patients (38%) tested positive for faecal nucleic acid, which was higher than previously reported.^9^, ^10^ Blood and urine samples of all patients were negative for SARS‐CoV‐2 nucleic acid, possibly because we included relatively few cases in this study and all of them were mild cases with common symptoms. The results of viral nucleic acid tests in the vaginal secretions of six female patients and the milk of one lactating patient were negative, consistent with previous studies.^11^ Similar to other reports, we found that the positive rate of viral nucleic acid test in respiratory tract specimens was the highest in the sputum, followed by nasal swab and oral swab.^12^ It is suggested that sputum collection should be given priority when collecting respiratory tract specimens of COVID‐19 patients for viral nucleic acid testing. In the absence of sputum, the nasal swab may be more sensitive than the oral swab for detection of SARS‐CoV‐2 nucleic acid.

In our study, two patients continued to test positive for SARS‐CoV‐2 nucleic acid in the faeces for up to 15 days after the negative results from respiratory tissues. Zhang et al^13^ reported that three children with COVID‐19 were discharged from the hospital with negative respiratory virus, and were re‐examined with positive nucleic acid from faeces within 10 days. Although the first symptoms of COVID‐19 are mainly respiratory symptoms, gastrointestinal symptoms were still found in many patients in clinical practice. Wang et al^14^ analysed 138 confirmed cases of COVID‐19 in China, in which gastrointestinal symptoms occurred in 10.12% of the cases. In another study, a total of 42 laboratory‐confirmed patients were enrolled, 8 (19.05%) of whom had gastrointestinal symptoms.^15^ Previous studies have confirmed that SARS‐CoV‐2 enters and infects host cells through the cell receptor angiotensin‐converting enzyme 2 (ACE2).^16^ Zhang et al^17^ found that ACE2 was highly expressed not only in alveolar epithelial cells, but also in oesophageal and lamellar epithelial cells as well as in epithelial cells in the ileum and colon. The above data suggest that SARS‐CoV‐2 may invade the digestive tract via ACE2 receptors, leading to gastrointestinal symptoms. Nouri‐vaskeh et al^18^ speculated on the existence of fecal‐oral transmission of SARS‐CoV‐2 by referring to published articles. Taken together, this highly suggests that faecal‐oral transmission may be another potential route for SARS‐CoV‐2. During the SARS epidemic in Hong Kong, China, there was a large‐scale community infection caused by the excrement of patients. A SARS patient with diarrhea visited a relative's home in a residential area and used the toilet twice. Finally, 321 people were infected and 42 died because of a flaw in the building's sewage system design.^19^ Therefore, based on the SARS prevention and control experience, we need to be cautious in discharging patients whose respiratory samples test negative for viral nucleic acid test but continue to show positivity in faeces, even though they have met the discharge criteria of the national “Diagnosis and Treatment Scheme of COVID‐19”, and protective measures will need to be enhanced to prevent faecal‐oral transmission. The author suggested that faecal virus nucleic acid should be tested as a routine monitoring index in the daily diagnosis and follow‐up of COVID‐19 patients, and discussed whether to increase the negative faecal nucleic acid test to two consecutive times in the discharge criteria.

Recurrence of viral nucleic acid in the sputum was shown in 4 of the 13 discharged COVID‐19 patients (30.7%) at a later follow‐up. The time interval for viral nucleic acid recurrence was 5 to 14 days. One of the patients experienced recurrence followed by a negative test result, which turned positive again at a later stage. The literature on nucleic acid positivity in patients with COVID‐19 after discharge is limited, with mostly case reports. On 27 February, Lan et al^20^ found that four patients all had positive results of the nucleic acid test of pharynx swabs in the re‐examination of four cured medical staff infected with SARS‐CoV‐2. But the proportion of patients with nucleic acid reactivation was not specified. On 25 February, at a press conference on the epidemic situation in Guangdong province, Song tie, the deputy director of the Guangdong provincial center for disease control and prevention (CDC), said that 14% of discharged patients tested positive for SARS‐CoV‐2 again in Guangdong province, China.^21^

There may be three possibilities for the recurrence of viral nucleic acid in discharged patients. One possibility is that the test results before discharge were false negative due to a flawed sample. It has been reported that the detection rate of viral nucleic acid in oral swabs is only 30–50% and is higher in nasal swabs. In comparison, the detection rate in the sputum is higher than that of both nasal and oral swabs.^12^ False negative results caused by improper sampling could be effectively reduced by collecting deep sputum and alveolar lavage fluid, increasing faecal examinations, and repeated testing of (suspected) patients. The second possibility is that the virus in the patient was not completely cleared, but the viral load was below the detection limit of the assay, resulting in the false negative results. With changes in the immune system of the host, the virus may re‐emerge and increase the viral load above the detection limit. Finally, cured patients may have developed a secondary infection with SARS‐CoV‐2 due to re‐exposure to the virus.

In our study, all four discharged patients had negative sputum(not pharyngeal swab) results in 4 to 5 consecutive viral nucleic acid tests with at least 1 day between each test before recurrence of the virus. This indicates that the results were unlikely to be false negative due to improper sampling. In an earlier study of COVID‐19, virus‐specific IgM and IgG antibody was detected in 50% (8/16) and 81% (13/16) of the patients’ serum, respectively, after 10 days of treatment. On day 15 after treatment, the presence of virus‐specific IgM and IgG was as high as 81% (13/16) and 100% (16/16), respectively, indicating that patients with COVID‐19 generally produce protective antibodies in the early stage of disease.^8^ A SARS‐CoV study showed that in SARS patients, specific IgG antibodies can persist for 12 years.^22^ These studies suggest that patients infected with SARS‐CoV‐2 carry protective antibodies after recovery and may be able to maintain immunity for a long time. And the four patients were kept in strict isolation at home after discharge, with no history of re‐epidemiological exposure. Therefore, the possibility of reinfection in a short time is very small. Simultaneously, we did not observe any clinical symptoms related to viral pneumonia during the follow‐up in the four patients with recurring viral nucleic acid and no progression of the disease was found in CT scans of the lungs, which indicated that the patients were not cases of recurrence of the disease but rather an asymptomatic virus carrier.

SARS‐CoV‐2 has a strong transmission capability and a fast infection rate. The elderly and those with chronic underlying diseases have a poor prognosis.^23^ At present, the sources of infection are COVID‐19 patients and asymptomatic virus carriers may also spread the virus. The results of this study indicated that under the current discharge criteria, there may be a large proportion of discharged patients who are still virus carriers. To determine whether these viral nucleic acid relapsing patients are infectious, we need to consider two factors. One is to clarify the relationship between the chance of viral transmission and viral load in patients. We can then determine the viral load in relapsing patients to assess their infectiousness. Second, it is necessary to distinguish whether the viral nucleic acid positivity of discharged patients is due to residual virus or active replication. Currently, these factors remain to be elucidated. Therefore, without clear evidence that discharged patients are not infectious, the patients themselves and others will be at great risk if they are not adequately followed‐up and strictly isolated after discharge.

## 5 CONCLUSION

In view of the current discharge criteria, the presence of viral nucleic acid in the faeces and the rate of recurrence in the sputum of discharged patients have led us to propose the following suggestions. First, it may be necessary to reassess the suitability of the current discharge criteria, which requires two consecutive days of negative viral nucleic acid testing in respiratory samples at least 1 day apart. Faecal virus nucleic acid should be tested as a routine monitoring index for COVID‐19, and it may be necessary to add a negative faecal result to the criteria. Second, medical institutions should strengthen the regular follow‐up and re‐examination of discharged patients and continuously monitor changes in viral nucleic acid in discharged patients. Considering the significance of this ongoing global public health emergency, although our conclusions are limited by the small sample size, we believe that the findings are important to understand SARS‐CoV‐2 relapse potential in COVID‐19 patients. In the next step, we will collect more data and evaluate the outcome of the disease in a larger population of discharged patients during a longer and more complete follow‐up period, providing a basis for optimizing discharge criteria and follow‐up management of discharged patients with COVID‐19.

## CONFLICT OF INTERESTS

The authors declare that there are no conflict of interests.

## AUTHOR CONTRIBUTIONS

All the authors participated in generating the idea. B.L. and G.H. took part in sample collection. Y.L., Y.W., and X.Z. tested all samples by RT‐PCR assay. Y.Y. took part in data collection and interpretation. Y.L. and Y.H. analysed and interpreted the data and wrote the manuscript. J.X. critically reviewed and edited it with his comments.

## Data Availability

With the permission of the corresponding authors, we can provide participant data without names and identifiers, but not the study protocol, statistical analysis plan, or informed consent form. Data can be provided after the article is published. Once the data can be made public, the research team will provide an email address for communication. The corresponding authors have the right to decide whether to share the data or not based on the research objectives and plan provided.

## References

[1] Huang C , Wang Y , Li X , et al. Clinical features of patients infected with 2019 novel coronavirus in Wuhan, China. Lancet. 2020;395(10223):497‐506. 31986264 10.1016/S0140-6736(20)30183-5 PMC7159299

[2] World Health Organization . Novel coronavirus‐China. 2020. http://www.who.int/csr/don/12-january-2020-novel-coronavirus-china/en/. Accessed January 19, 2020.

[3] World Health Organization . Corona virus disease (COVID‐19) outbreak. 2020. https://www.who.int. Accessed March 15, 2020.

[4] National health commission of the People's Republic of China . Update on the novel coronavirus pneumonia outbreak. 2020. http://www.nhc.gov.cn/xcs/yqtb/202003/8721a8bc007b448db32489ea74b321fc.shtml. Accessed March 28, 2020.

[5] Lu R , Zhao X , Li J , et al. Genomic characterisation and epidemiology of 2019 novel coronavirus: implications for virus origins and receptor binding. Lancet. 2020;395(10224):565‐574. 32007145 10.1016/S0140-6736(20)30251-8 PMC7159086

[6] National health commission of the People's Republic of China . Diagnosis and treatment scheme of novel coronavirus infected pneumonia (trial version 5). 2020. http://www.gov.cn/zhuanti/2020-02/09/content_5476407.htm. Accessed February 9, 2020.

[7] Xia J , Tong J , Liu M , Shen Y , Guo D. Evaluation of coronavirus in tears and conjunctival secretions of patients with SARS‐CoV‐2 infection. J Med Virol. 2020;92(6):589‐594. 10.1002/jmv.25725 32100876 PMC7228294

[8] Zhang W , Du RH , Li B , et al. Molecular and serological investigation of 2019‐nCoV infected patients: implication of multiple shedding routes. Emerg Microbes Infect. 2020;9(1):386‐389. 10.1080/22221751.2020.1729071 32065057 PMC7048229

[9] Guan W‐J , Ni Z‐Y , Hu Y , Liang W‐H , Ou C‐Q , He J‐X , et al. Clinical characteristics of coronavirus disease 2019 in China. N Engl J Med. 2020;382(18):1708‐1720. 10.1056/NEJMoa2002032 32109013 PMC7092819

[10] Zhang J , Wang S , Xue Y. Fecal specimen diagnosis 2019 novel coronavirus‐infected pneumonia. J Med Virol. 2020;92(6):680‐682. 10.1002/jmv.257422 32124995 PMC7228355

[11] Chen H , Guo J , Wang C , et al. Clinical characteristics and intrauterine vertical transmission potential of COVID‐19 infection in nine pregnant women: a retrospective review of medical records. Lancet. 2020;395(10226):809‐815. 10.1016/s0140-6736(20)30360-3 32151335 PMC7159281

[12] Chan JF‐W , Yuan S , Kok K‐H , et al. A familial cluster of pneumonia associated with the 2019 novel coronavirus indicating person‐to‐person transmission: a study of a family cluster. Lancet. 2020;395(10223):514‐523. 31986261 10.1016/S0140-6736(20)30154-9 PMC7159286

[13] Zhang T , Cui X , Zhao X , et al. Detectable SARS‐CoV‐2 viral RNA in feces of three children during recovery period of COVID‐19 pneumonia. J Med Virol. 2020;92(7):909‐914. 10.1002/jmv.25795 32222992 PMC7228213

[14] Wang D , Hu B , Hu C , et al. Clinical characteristics of 138 hospitalized patients with 2019 novel coronavirus–infected pneumonia in Wuhan, China. JAMA. 2020;323(11):1061‐1069. 32031570 10.1001/jama.2020.1585 PMC7042881

[15] Chen Y , Chen L , Deng Q , et al. The presence of SARS‐CoV‐2 RNA in the feces of COVID‐19 patients. J Med Virol. 2020;92(7):833‐840. 32243607 10.1002/jmv.25825

[16] Hou P , Yang X‐L , Wang X‐G , et al. Discovery of a novel coronavirus associated with the recent pneumonia outbreak in humans and its potential bat origin. BioRxiv. 2020. 10.1101/2020.01.22.914952

[17] Zhang H , Kang Z , Gong H , et al. Digestive system is a potential route of COVID‐19: an analysis of single‐cell coexpression pattern of key proteins in viral entry process. Gut. 2020;69(6):1010‐1018.

[18] Nouri‐Vaskeh M , Alizadeh L. Fecal transmission in COVID‐19: a potential shedding route [published online ahead of print April 1, 2020]. J Med Virol. 2020. 10.1002/jmv.25816 PMC7228240 32239515

[19] Peiris JS , Chu CM , Cheng VC , et al. Clinical progression and viral load in a community outbreak of coronavirus‐associated SARS pneumonia: a prospective study. Lancet. 2003;361(9371):1767‐1772. 10.1016/s0140-6736(03)13412-5 12781535 PMC7112410

[20] Lan L , Xu D , Ye G , et al. Positive RT‐PCR Test Results in Patients Recovered From COVID‐19 [published online ahead of print, 2020 Feb 27]. JAMA. 2020;323(15):1502‐1503. 10.1001/jama.2020.2783 32105304 PMC7047852

[21] The 31st press conference on epidemic prevention and control from Guangdong Provincial Government Information Office. 2020. http://gdio.southcn.com/g/2020-02/25/content_190429333.htm. Accessed February 2, 2020 (in Chinese).

[22] Yang Y , Yang MH , Shen CG , et al. Evaluating the accuracy of different respiratory specimens in the laboratory diagnosis and monitoring the viral shedding of 2019‐nCoV infections. medRxiv. 2020. 10.1101/2020.02.11.20021493

[23] Kui L , Fang Y‐Y , Deng Y , et al. Clinical characteristics of novel coronavirus cases in tertiary hospitals in Hubei province. Chin Med J. 2020. 10.1097/CM9.0000000000000744 PMC7147277 32044814

